# Returning to normal? ‘Building back better’ in the Dominican education system after Tropical Storm Erika and Hurricane Maria

**DOI:** 10.1111/disa.12536

**Published:** 2022-05-19

**Authors:** Martin Parham

**Affiliations:** ^1^ Lecturer University of Central Lancashire, School of Natural Sciences United Kingdom

**Keywords:** build back better, child‐centred disaster risk reduction (CCDRR), comprehensive school safety framework, Dominica, education, Hurricane Maria

## Abstract

Child‐centred disaster risk reduction aims to reduce child vulnerability and increase resilience to disasters. The 2015 Comprehensive School Safety Framework (CSSF) sought to decrease hazard risks to education. Between 2015 and 2017, Dominica was struck by Tropical Storm Erika and Hurricane Maria, which significantly affected the education system at the local and national scales. Since Maria, a couple of national initiatives (Safer Schools and Smart Schools) have been introduced to increase resilience and meet the CSSF's objectives. This paper assesses progress made through a qualitative analysis of interviews with 29 school leaders, government officials, and disaster risk reduction stakeholders. Implementation of the climate resilience programme in 2018 resulted in nationwide teacher training and production of school disaster plans. Limited successes have improved social resilience, but short‐term implementation due to COVID‐19 and a lack of a teacher knowledge base have presented challenges to the scheme's long‐term sustainability and the implementation of the CSSF's goals.

## Introduction

### Concepts of disaster risk education and child‐centred disaster risk reduction

The premise of disaster risk reduction (DRR) is ‘the concept and practice of reducing disaster risks through systematic efforts to analyse and manage the causal factors of disasters, including through reduced exposure to hazards, lessened vulnerability of people and property, wise management of land and the environment, and improved preparedness for adverse events’ (UNISDR, 2009, pp. 10–11). Education helps to achieve this through improved understanding of disaster risk, leading to more resilient and less vulnerable societies (UNISDR, 2017). Educational approaches to DRR in communities are varied and may include media campaigns, public information initiatives, and community‐based training. School education, though, provides a focused opportunity to develop both student understanding, awareness, decisionmaking, and action, helping to build bottom‐up community resilience (Cox et al., 2017; Bandecchi et al., 2019).

Parham et al. (2021) summarise the misrepresentation of children in DRR studies. Children are considered to be passive receivers of information (Mitchell et al., 2008; Amri et al., 2017a, 2017b) or unable to make decisions (Hart, 1992), which impact on their safety. However, the 1989 United Nations Convention on the Rights of the Child (CRC) (United Nations General Assembly, 1989) shows that children have the right to be protected and safeguarded and to have a voice in matters that affect them. They should also have a right to know how to stay safe in the context of their home, school, and community (UN, 1989).

The study of child‐centred disaster risk reduction (CCDRR), defined as ‘[r]ecognising and drawing on the rights, needs and capacities of children in reducing risk and enhancing the resilience of communities and nations’ (Hore et al., 2018, p. 1), has attempted to fill this gap in understanding. Newham et al. (2019) highlight the need for adolescents to be participants in disaster recovery, but they note that the practical application of this is unclear. Various authors (Ronan and Johnston, 2001; Wisner, 2006; Tanner and Haynes, 2009; Tanner, 2010; Ronan, Crellin, and Johnston, 2012; Wisner, Galliard, and Kelman, 2012; Ronan, 2015; Mutch, 2015; Ronan et al., 2016; Delicado, Rowland, and Fonseca, 2017; Wisner et al., 2018; Sakurai et al., 2018; Morais, 2019) have promoted the benefits of integrating CCDRR into schools and the education curriculum. These benefits include an integrated CCDRR curriculum to reinforce learning, developing simulation exercises to encourage participation, and making the school a focal point to develop psychosocial practices with students.

CCDRR has grown in importance since the 1990s (Ronan, 2001) and became a priority in the Hyogo Framework for Action 2005–2015 (HFA) (UNISDR, 2005). However, Ronan et al. (2016) underlined the gaps in CCDRR, specifically, a need for a multi‐hazard focus and to adopt a skills or action‐based approach instead of just knowledge enhancement (Parham et al., 2021). Several authors (Johnson et al., 2014; Haynes and Tanner, 2015, Ronan et al., 2016; Parham et al., 2021) recognise that a long‐term assessment of CCDRR programmes is needed, during and following hazard events, to understand the effectiveness of CCDRR in the education curriculum.

### International DRR educational policy

Priority 3 of the HFA identified specific actions to promote DRR for education at an international scale. Notably:

- to include DRR knowledge in relevant areas of the school curriculum;
- to perform a risk assessment of local risk and preparedness in educational institutions; and
- to implement programmes to improve learning to minimise the effects of hazards (UNISDR, 2005).

From 2005, annual United Nations (UN) Global Platform for Disaster Risk Reduction meetings served to reinforce the aims of integrating CCDRR into education curricula by 2015 (UNISDR, 2015). Although education was activity promoted at the Third Session of the Global Platform on 8–13 May 2011, a deadline to integrate DRR into the school curriculum had disappeared by 2013 (UNISDR, 2013). This downgrading of CCDRR education was in part due to a difficulty in implementing it in national curricula, despite one‐half of countries reporting to the HFA that DRR education was included in the national curriculum (Ronan, 2015).

The Sendai Framework for Disaster Risk Reduction 2015–2030 (SFA) built on the principles of the HFA and sought to achieve a ‘substantial reduction of disaster risk and losses in lives, livelihoods and health and in the economic, physical, social, cultural and environmental assets of persons, businesses, communities and countries’ (United Nations, 2015, p. 12). However, unlike the HFA, the role of education was not explicit in the SFA's priorities for action. Instead, the SFA included seven ‘related’ targets that could be applied implicitly to the education sector (see Table 1).

**Table 1:** SFA ‘related targets’ for the educator sector

The SFA's seven global targets	Link to schools/education
1. ‘Substantially reduce global disaster mortality by 2030, aiming to lower the average per 100,000 global mortality rate in the decade between 2020–2030 compared to the period 2005–2015’.	Minimise the number of deaths and injuries due to hazard impacts on schools.
2. ‘Substantially reduce the number of affected people globally by 2030, aiming to lower the average global figure per 100,000 in the decade between 2020–2030 compared to the period 2005–2015’.	Substantially reduce the number of school children affected by disaster impacts of all sizes.
3. ‘Reduce direct disaster economic loss in relation to global gross domestic product (GDP) by 2030’.	Reduce education sector investment losses due to hazard impacts.
4. ‘Substantially reduce disaster damage to critical infrastructure and disruption of basic services, among them health and educational facilities, including through developing their resilience by 2030’.	Minimise school days lost due to hazard impacts.
5. ‘Substantially increase the number of countries with national and local disaster risk reduction strategies by 2020’.	Countries have education sector risk reduction strategies.
6. ‘Substantially enhance international cooperation to developing countries through adequate and sustainable support to complement their national actions for implementation of the present Framework by 2030’.	Countries work together to achieve comprehensive school safety.
7. ‘Substantially increase the availability of and access to multi‐hazard early warning systems and disaster risk information and assessments to people by 2030’.	Schools have access to and use early warning systems.

**Source**: authors and United Nations (2015, p. 12).

The Comprehensive School Safety Framework (CSSF) was created in 2015 to follow on from the Worldwide Initiative for Safe Schools and the Global Alliance for Disaster Risk Reduction and Resilience in the Education Sector. It aimed to adopt a multi‐hazard approach, improve child participation, strengthen resilience through education, and allow for the continuity of education through hazards (UNISDR, 2017). It sought to integrate the SFA's seven global targets (see Table 1) into the education sector, using three pillars: (i) safe learning facilities; (ii) school disaster management; and (iii) risk reduction and resilience education (UNISDR, 2017). Under these pillars, the CSSF established four broad aims: (i) to improve global leadership on disaster risk education (DRE) and resilience in education and to promote comprehensive school safety (CSS); (ii) to enhance safe learning facilities to provide guidance and to assess educational infrastructure and facilities to create safe school environments; (iii) to foster school disaster and emergency management of multi‐hazard risk, for both students and local communities; and (iv) to develop risk reduction and resilient education.

Without explicit reference to education priorities, as in the HFA, the CSSF outlined a sustainable pathway for developing school safety and integrating CCDRR into the school curriculum, thereby creating a plan for countries to develop resilient students and communities. However, the CSSF did not set targeted deadlines for action. States were encouraged to forge safe infrastructure, adopt a multi‐hazard philosophy within education, and encourage partnerships between local schools and with DRR stakeholders at the regional and national level. Paci‐Green et al. (2020) report that after the CSS global baseline survey in 2017, only 65 per cent of reviewed countries had included DRR and resilience in national curricula and that only 25 per cent of schools gave teachers specific training. Only 31 per cent of countries had funded a hazard risk assessment of school building stock, while only 37 per cent of countries had limited the use of schools as education shelters. Only 18 countries in Latin America and the Caribbean responded to the survey; only four of the 18 were in the Caribbean (Paci‐Green et al., 2020).

This paper assesses the extent to which one small island developing state in the Caribbean, Dominica, has managed to integrate the CSSF through a longitudinal analysis. Dominica is a suitable case study because the documented changes to DRE have occurred over a time period when significant disaster events—Tropical Storm Erika in 2015 and Hurricane Maria in 2017—have impacted the island. Drawing on stakeholder interviews, this paper evaluates the degree to which CSSF implementation has taken place and identifies the opportunities presented and the challenges faced.

## Case study of disaster risk in Dominica: context

### Historical background

Dominica is a Caribbean Island in the Lesser Antilles (15°N and 61°W). It is classified as an upper middle‐income country and has a population of approximately 71,000 (World Bank, 2018). Its proximity to the Lesser Antilles Trench (LAT) and its location in the western Atlantic Ocean result in high exposure to both geophysical and hydrometeorological hazards (Benson et al., 2001; Lindsay et al., 2005; Honychurch, 2017; Barclay et al., 2019).

Dominica's history has made it vulnerable to natural hazards. Its colonial past created a dependence on monoculture, notably coffee, sugar, cocoa, limes, and bananas, which have, at one point in time, dominated as the main export crop (Barclay et al., 2019). Attempts to diversify have been superseded by capitalism and colonialism, resulting in a weakened economy (Honychurch, 1995). The historical approach to disaster response has varied, from colonial loans or grants to repairs, rather than ‘building back better’ (Honychurch, 2017). Despite having had a draft National Disaster Plan since 2001 (Government of the Commonwealth of Dominica, 2001), there is a continued reliance on international aid and official development assistance (ODA) (Grydehoj and Kelman, 2020), as well as internal government budgetary reallocations and consultancy, leading to inefficiencies in response and recovery (Barclay et al., 2019).

### Vulnerability and institutional capacity in Dominica

Dominica is a multi‐hazard environment and is vulnerable to a range of natural hazards, most particularly tropical cyclones in the hurricane season between August and November. Coastal and riverine populations are vulnerable to river flooding, storm surges, and wind damage (Honychurch, 2017). The island's situation close to the LAT exposes it to annual seismic events and potential tsunami and volcanic activity, emanating from one of the nine active centres (Lindsay et al., 2005). Topographic and climatic factors also make landslides, bush fires, flooding, and storm surges regular occurrences (Benson et al., 2001).

Dominica was ranked fifth on the World Bank's 2005 list of Natural Disaster Hotspots—that is, countries with a relatively high mortality risk owing to multiple hazards (World Bank, 2005). In a recent report, it was classified as the fourth most vulnerable of the Caribbean states to climate hazards (Government of the Commonwealth of Dominica, 2020).

Despite the vulnerability, Dominica did not implement specific disaster risk management legislation until after 2011 (Carby, 2011) (see Table 2). It is reliant on geophysical monitoring provided by data from the Seismic Research Council (Dondin et al., 2019), while hydrometeorological forecasting is provided by the Dominica Meteorological Service from six stations, supplemented by information from the National Oceanic and Atmospheric Administration (NOAA) (Government of the Commonwealth of Dominica, 2022). In 2011, a United Nations Development Programme report (Carby, 2011) outlined a series of constraints faced by Dominica in its response to DRR (see Table 2).

**Table 2:** Recognised constraints to disaster risk management in Dominica

• A lack of a necessary legislative framework.
• Insufficient central government support.
• Inadequate support for capacity‐building (before Hurricane Maria).
• Not enough coordination and cooperation among sectors, including government, private, and civil society bodies, to inform the development process.
• The absence of a communications platform, which is common to the strategies of the HFA.
• No champion to promote the adoption and implementation of the HFA in Dominica.
• An inconsistent approach to education and simulation programmes.

**Source**: Carby (2011).

Recent hazard risks in Dominica are well documented (Honychurch, 1995; Lindsay et al., 2005; Van Westen, 2016; Barclay et al., 2019). Hurricane David (1979) and Hurricane Maria (2017) represent the largest magnitude events to affect Dominica, whereas Tropical Storm Erika (2015) and Hurricane Maria (2017) had the greatest impact on gross domestic product (GDP) (Barclay et al., 2019; Government of the Commonwealth of Dominica, 2020).

### Tropical Storm Erika

Tropical Storm Erika passed more than 150 kilometres to the north of Dominica on 27 August 2015. It had no obvious central circulation but had embedded gyres within the broader circulation, one of which formed over Dominica, leading to a high amount of rainfall amassing over the island between 08:00 and 18:00 UTC (Coordinated Universal Time) (Pasch and Penny, 2015). Ogden (2016) reported that this resulted in 300–750 millimetres of precipitation falling in a four‐hour period, with a peak runoff rate of 80 per cent of peak rainfall. Although the storm's track and formation were anticipated by the National Hurricane Center in the United States, the high levels of rainfall that developed over Dominica were not (Pasch and Penny, 2015). The disaster event cost the island USD 483 million, equivalent to 90 per cent of its GDP (World Bank, 2015), and claimed the lives of 30 people (Pasch and Penny, 2015), as well as leaving 574 homeless. The most significant damage was to the southern coastal parts of Dominica; the village of Petite Savanne suffered the worst. The key issue for Dominica was a lack of interpretative capacity of available NOAA data, owing to the absence of awareness of the perceived impacts of the storm tracking north of Guadeloupe (Pasch and Penny, 2015).

### Hurricane Maria

Hurricane Maria was one of the five costliest tropical hurricanes to hit the Caribbean at that time, inflicting USD 63 million in overall losses (of which USD 30 million were insured, mainly in US territories) and causing 108 deaths in the region (Wilkinson, Twigg, and Few, 2018). Maria formed 580 miles east of Barbados on 16 September 2017 and tracked west until 17 September, when it intensified rapidly. By 18 September, it had become a 1,000‐knot hurricane; as it neared Dominica, it became a Category 5 hurricane with 145‐knot winds and an estimated central pressure of 922 millibars (Pasch, Penny, and Berg, 2019). It is the strongest hurricane on record to make landfall in Dominica.

The island received huge amounts of rainfall, with a maximum of 22.8 inches, resulting in serious flooding and mudslides (Pasch, Penny, and Berg, 2019). The storm affected 100 per cent of the population, with the greatest damage occurring in slope and coastal locations. Across Dominica, Maria killed 31 people directly and left 34 missing, resulting in USD 1.31 billion of total damage (Pasch, Penny, and Berg, 2019). This event led to Prime Minister Roosevelt Skerrit declaring that Dominica should endeavour to become the world's first ‘climate resilient nation’ (Government of the Commonwealth of Dominica, 2020).

### DRR education in Dominica

Education in Dominica is compulsory between the ages of 5 and 16. Students attend primary school until the age of 11 and then secondary school between the ages of 11 and 16. At secondary school, they study for Caribbean Examinations Council qualifications. They have a wide curriculum in Forms 1–3, between the ages of 11 and 14. At 14, in Form 4, students undertake a two‐year programme of study for the Caribbean Secondary Education Certificate (CSEC). After secondary school, they study for the Caribbean Advanced Proficiency Examination (CAPE) at the State College, located in Roseau. Many of them remain in education after the CAPE, leaving the island to study at The University of the West Indies on Barbados, Antigua, or Trinidad, or travel further afield to enter the university system in the US.

Students leaving State College can attend teacher training college and then are given the opportunity to work in schools, allocated by the government. Some schools only employ teachers with a university degree; however, this is not a requirement of the Ministry of Education.

Despite a regional commitment to integrate DRE into formal education, institutional commitment has been weak (Serrant, 2013; Knight, 2015). Until 2017, students studied a curriculum with some links to DRR activities, during social science classes (Forms 1–3, first three years of secondary education). Formally, the study of natural hazards is part of the geography CSEC, a course that educates students about natural hazards across the Caribbean and in a wider context, with a heavy focus on atmospheric hazards. Between 20 and 30 per cent of pupils study geography at CSEC level (Parham et al., 2021). Implementation of DRE was dependent on teacher input until 2017–18 when the government initiated its Climate Resilience and Recovery Plan 2020–2030 (Government of the Commonwealth of Dominica, 2020). The role of the Ministry of Education, as outlined by the National Disaster Plan (Government of the Commonwealth of Dominica, 2001), was to initiate educational programmes to help schools prepare students for an impending disaster, principally the maintenance of schools as disaster shelters or the organisation of drills for a fire or earthquake. The National Disaster Plan (Government of the Commonwealth of Dominica, 2001) does not reference the provision of DRE content or simulations of multi‐hazard risk in the curriculum. Consequently, until the disasters of 2015–17, student preparation for hazard events was principally based on the past experiences of teaching staff (Parham et al., 2021). The fact that a 2021 Regional Action Plan for DRR in the Americas and the Caribbean suggested a need to incorporate hazard knowledge and disaster risk information into the school curriculum indicates that improvements in DRE can still be made (UNDRR, 2021).

Schools also play a role in disaster response, since all of them are in areas that face at least one disaster risk (Paul‐Rolle, 2014). After Hurricane David in 1979, 59 schools were damaged and had to be rebuilt with concrete roofs. Since 1980, schools have undergone limited rehabilitation; a programme to retrofit 20 schools was announced in 1998 (Government of the Commonwealth of Dominica, 1998). Little additional work was undertaken, however, until after Hurricane Maria. Despite recommendations to reduce the number of schools being used as shelters, 17 were still serving that purpose in 2017 (of 135 hurricane shelters available), most of them secondary schools (UNICEF Eastern Caribbean, 2017).

## Methods

To understand how the education system has responded to disasters in Dominica, qualitative research was conducted longitudinally between 2014 and 2018. This paper also draws on research carried out in Dominica between 2013 and 2018, looking at the impact of DRR on secondary school students’ perceptions and education (Parham et al., 2021).

There are seven state schools in Dominica and eight private or assisted schools operating as secondary educational facilities. The Ministry of Education gave the author permission to work with five of these schools. Their location is in or near to the three largest urban areas of Dominica: the capital city, Roseau (southwest); Portsmouth (northwest); and Marigot (east coast). Secondary schools were chosen as the study focus as they represent a larger sociodemographic mixture of population and areas with unique multi‐hazard risks (Parham et al., 2021).

Data collection aimed to yield insights into the changing perspectives of senior leaders or teachers from the five allocated secondary education institutions and of stakeholders involved in the delivery of educational DRR. A total of 29 participants (5 school principals, 14 teachers, 3 NGO (non‐governmental organisation) staff, 5 government department staff, and 2 academic researchers) agreed to contribute to the study. Although this is a small sample, it reflects the locally available expertise. More than 119 students from the sample schools also contributed qualitative perceptions based on responses to PRISM (Pictorial Representation of Illness and Self Measure) surveys conducted in 2013–18 (Parham et al., 2021).^1^ A longitudinal approach was selected for this study to understand change better (Siegrist, 2013). Stakeholder interviews were held with the same respondents over the study period, 2013–18, mainly in October and April, to comprehend pre‐ and post‐hurricane season perspectives. Visits before 2015 helped to reveal the workings of DRE in the education curricula and changes to approaches. Visits in 2016 sought to gauge the impacts of Tropical Storm Erika. And data collected after 2017 aimed to determine the impacts of Hurricane Maria on educational facilities and the changes introduced through the implementation of the government's climate resilience policy.

Qualitative data, from DRR stakeholders, were gathered using semi‐structured interviews with open‐ended questions (Williams and Webb, 2021). These were based on three themes: (i) the implementation of DRE before 2015; (ii) the impacts on schooling and educational programmes after Tropical Storm Erika and Hurricane Maria; and (iii) attempts to integrate DRR into education and improve school student's resilience.

Follow‐up interviews online or sometimes via e‐mail were conducted with the study participants in 2021 to evaluate initiatives to incorporate DRR in schools. All of the interviews were transcribed, recorded, and coded using principles outlined in Bryman (2016). A deductive method was employed with the DRR experts to code the transcribed interviews (Sayer, 1992). All references to the names and roles of interviewees have been anonymised, and ethical approval of the study was granted by the University of Portsmouth in the United Kingdom. The Ministry of Education and school principals allowed the author to conduct educational sessions with the students, owing to his qualified teacher status (Parham et al., 2021).

## Analysis: post‐disaster educational change in Dominica

### Impacts of Tropical Storm Erika on education

Tropical Storm Erika claimed the equivalent of 90 per cent of Dominica's GDP, causing USD 3.5 million in damage to education (Collymore et al., 2017). It directly affected 23 schools, damaging 13 and destroying two across eight parishes. The start of the academic year was pushed back from 1 to 14 September for schools used as shelters or damaged; by 28 September, all schools had been reopened (Government of the Commonwealth of Dominica, 2015). Tropical Storm Erika cost XCD 10.7 million to correct: XCD 9.5 million in repairs and rebuilding and XCD 1.2 million in non‐structural costs, including psychosocial support in the form of the Return to Happiness (RTH) initiative (Government of the Commonwealth of Dominica, 2015).

Erika exposed problems for education. Damage to the cross‐country and coastal road infrastructure hindered some teachers’ capacity to return to work, while extensive landslide activity impacted telecommunications, limiting options for online learning. One teacher said: ‘Erika only affected my journey to school as some bridges collapsed and roads collapsed’.

Questions regarding the safety of ageing school infrastructure led to a need to develop safe schools with access to clean water and sanitation and limit structural damage, particularly in seriously affected communities in the south (Government of the Commonwealth of Dominica, 2015). While it was clear that the effects were spatially variable, teaching staff commented on their frustration at not preparing the students adequately because of its unanticipated arrival. One principal stated that ‘the storm was unexpected, and so we did not give the students any warning’. Another added that ‘the storm occurred during the night, meaning that we were not able to use the school as [a] focal point to keep the children safe’. Teachers in Roseau noted that schooling was temporarily affected and that the children from the southern part of the island suffered the greatest impact due to a lack of access or the direct ramifications of the storm.

Post disaster, schools were encouraged by the Ministry of Education to update their hazard policies and continue to practise drills. Implementation of these changes varied depending on the location of the affected school. There was mixed feeling within government about the consequences of Erika for the education system. One government official remarked: ‘Tropical Storm Erica has changed the landscape and made people more aware, however it is not clear that we have improved our readiness for another storm hazard’. Another pointed out that ‘post Erika communication between government departments and parents was updated through the use of Moodle on the Ministry of Education website’.

However, the introduction of psychosocial training and the additional welfare assistance given to students proved to be a successful pilot project and led to larger‐scale implementation after Hurricane Maria. The initiation of the RTH initiative of the United Nations Children's Fund (UNICEF) allowed teaching staff to develop psychosocial training to help affected students share their emotional experiences on their return to school. The RTH programme sought to help children deal with the trauma through creative activities. As of 2018, it had reached 17,000 children and trained 1,500 facilitators (Knight, 2018). One teacher's comments demonstrate the positive feelings towards the scheme: ‘the psychosocial training was hugely beneficial to students who had been affected and allowed them to share their emotions and losses’.

Tropical Storm Erika exposed the variability of DRE in the school curriculum. Several students from Roseau and the east coast said that they were not aware of the nature of flood damage caused by heavy rainfall and that they did not clearly understand the links between intense rainfall and landslide hazards. The unexpected nature of the event and the time of day of its occurrence presented a challenge to DRR stakeholders on the island.

After the event, a change in the running of the Office of Disaster Management shifted attention towards improving national policy rather than continuing with existing bottom‐up community‐led schemes. Plans to move affected residents and repair damage were enacted as Pointe Dublique and Petite Savanne were categorised as ‘special disaster areas'; people were moved to a new housing development at Bellevue Chopin (UNDP, 2020). This project highlighted the key priorities at the time: transport; housing; and agriculture (ACAPS, 2017). However, there was a recognised need to build community capacity, through training as first responders and education (ACAPS, 2017). Whole island changes to DRE were not implemented at this juncture.

### Impacts of Hurricane Maria on education

Hurricane Maria caused a wide range of impacts across all communities of Dominica. School building stock was a focus of both storm damage and damage caused by inhabitation. Of the 73 primary and secondary schools, only four suffered no damage, whereas all 15 secondary schools were damaged, five significantly and nine partially (Government of the Commonwealth of Dominica, 2017). The effects of this storm led to complete closure of schools and colleges for at least one month; some students did not fully return to school for seven months. One principal said that: ‘some schools reopened in late October with students back in November, but some schools still had families staying in them [as shelters]’.

The scale of Maria presented Dominica with much greater issues than had previously been the case. The rapid increase in the intensity of the hurricane, from Category 1 to 5, and its changing track towards the island, meant that relative preparation time was limited (Pasch, Penny, and Berg, 2019). The structural damage to schools took communities by surprise. The island‐wide coverage of the hurricane resulted in a need for humane living conditions; schools suffered, therefore, as they continued to be used as shelters (ACAPS, 2018). In all of the five schools studied, principals reported either looting and/or structural failures, which limited the normal resumption of activities. The loss of telecommunications across the island (ACAPS, 2018) meant that online learning was not possible in some communities, resulting in inequality in education received between year groups and within communities.

Normality did not return to schools until after April 2018, following a staggered return. Schools operated at limited capacity between October 2018 and April 2019, prioritising those students with CSEC examinations and restricting the school day to four hours.

School principals noted that the challenges of reopening schools ranged from unsafe infrastructure to fluctuating school rolls. One principal said: ‘schools suffered damage to roofs and structural damage, resulting in an inability to provide capacity for students’. Another added that ‘there were fluctuations in numbers on roll as some had left the island’.

Despite these challenges, another principal underlined how the admission policy was changed for student families forced to move to access education: ‘we allowed students from other schools to temporarily register’. This allowed affected students unable to travel long distances to receive education from the nearest institution.

After reopening, schools were able to offer psychosocial support through cognitive behavioural therapy, as part of the Child Friendly Spaces and RTH initiatives. Teachers felt comfortable in delivering the assistance as they had received training after Tropical Storm Erika. The introduction of psychosocial support through the RTH initiative did allow for the gradual reintegration of students as they shared experiences with trained teaching staff. One teacher stated that: ‘The Ministry [of Education] organised three days of non‐teaching when students returned for psycho‐social support so that students received coping mechanisms to deal with their experiences’. However, various teachers commented on the insufficient teaching of DRE prior to Hurricane Maria. One emphasised that: ‘Students were not helped by our teaching of hazard prior to the event’.

Until Maria, the national curriculum did not make explicit reference to DRR, but it did include fire and earthquake drills and teaching about the impacts of hurricanes. Students commented on how their lack of experience of a Category 5 hurricane left them unprepared for the extensiveness of the damage caused by flood water and landslides across the island. Knowledge of an event on the scale of Maria, not experienced since Hurricane David in 1979, had skipped generations, leading to gaps in awareness and preparation.

Within 12 months of Maria, the government had initiated its short‐term priority to ‘build back better', which included a damage assessment of educational facilities, as part of the Safer Schools initiative. Schools that sustained damage due to the hurricane benefitted from temporary teaching accommodation provided by international agencies such as UNICEF and NGOs such as Samaritan's Purse, and targeted tutoring was offered to support at‐risk students. These approaches allowed education to return quicker, even while some schools were still being used as shelters. They also showed the value of the support of international actors for education and the reliance of small island developing states on outside assistance.

Post Maria, the government recognised the need to integrate DRR into the education system by improving training for students and teachers to promote hazard awareness to increase resilience (Government of the Commonwealth of Dominica, 2017). This was intended to develop both understanding and training, addressing the gaps in provision highlighted by Ronan et al. (2016).

### Educational change in response to Hurricane Maria

In the immediate aftermath of Maria, Prime Minister Skerrit informed the United Nations General Assembly that Dominica was an ‘international humanitarian emergency’ and promoted a vision to make Dominica the first ‘climate resilient nation', leading to the National Resilience Development Strategy 2030 (IFRC, 2021). The CRRP was developed to implement the strategy and had three aims, based on: (i) climate‐resilient systems; (ii) prudent disaster risk management; and (iii) effective disaster risk response and recovery (Government of the Commonwealth of Dominica, 2020). Education did not feature directly in the CRRP. It featured implicitly in the ‘Strong Communities’ goal and improvements to school infrastructure was only included as one of the 20 climate resilience targets, to be achieved by 2030 (Government of the Commonwealth of Dominica, 2020). Target 14 stipulated: ‘[n]o more than 5 per cent of schools and healthcare facilities severely damaged or destroyed by an extreme weather event’ (Government of the Commonwealth of Dominica, 2020, p. iii). This was a positive indicator of improving school infrastructure to boost resilience to climate hazards, but it did not account for the potential geophysical impact. The CRRP set out 10 critical high‐impact climate resilient initiatives, yet only one of them, the Resilient Dominica Physical Plan, explicitly addressed education, but with a focus on infrastructure. There was still a need to integrate DRR into the school curriculum.

The government proposed an updated national curriculum in 2018 to include DRR and to train teachers in DRR and emergency readiness. An intermediate aim to reduce the proportion of schools serving as long‐term shelters (10 per cent by 2030) centred on the issue of losing school days to accommodate climate refugees. To achieve its objectives, the government initiated two nationwide schemes: (i) Smart Schools, to improve technological capacity in schools; and (ii) Safer Schools, to improve youth resilience (part of the Child Friendly Spaces initiative).

The Child Friendly Spaces initiative sought to ‘promote a child centred approach to learning and is concerned with the health, safety security, nutritional status and psychological well‐being of the child as well as teacher training and appropriateness of teaching methods and learning resources’ (Government of the Commonwealth of Dominica, 2017). This was a positive action that aimed to tie in with the student‐centred approach to integrating DRR into schools, as outlined by numerous authors (Benson and Bugge, 2007; Peek, 2008; Haynes and Tanner, 2015; Sharpe, 2009; Kagawa and Selby, 2012; Selby and Kagawa, 2012; Sakurai et al., 2015; Ronan et al., 2016).

Schools were asked to produce their own bespoke hazard plans and to appraise the hazards affecting their site. One government official said: ‘We organised a pilot scheme in schools to get students to assess the impact of natural hazards in their school’. School principals noted that this had to involve face‐to‐face training involving both staff members and students. As a result, one government official underlined that: ‘schools had to complete a DRR booklet and submit [it]—all 73 schools must do this … to assess their own vulnerabilities’.

An IsraAID official involved in the scheme stated that ‘we aim to come up with tailor‐made [DRR] scenarios for each school’ through ‘school staff developing DRR with children, creating [a] student‐led initiative’. All schools were expected to have a disaster plan by July 2018. The same official commented that the aim was to ‘develop DRR in education through either “infusion” or by adding another topic [subject] in the curriculum', with students engaging ‘at three times in their education: at three to five years; [in] fourth grade; and [in the] third form’.

This approach would address some of the issues faced during Hurricane Maria, as it would allow school managers to identify deficiencies on their own campuses and work towards establishing plans to deal with multi‐hazard risks. In addition, it would fulfil the key aims of the CSSF initiative (UNICEF Eastern Caribbean, 2017) and build on recommendations to develop capacity (ACAPS, 2018). Tackling this matter further by integrating DRR into the school curriculum was an innovative step that would begin to address community vulnerability on a local scale.

Two staff from each school were trained in multi‐hazard risks facing Dominica by IsraAID and in emergency training through the Office of Disaster Management's Community Emergency Response Training (CERT) scheme. Emphasis was placed on these teachers disseminating information and contributing to the creation of the bespoke school plans for infrastructural improvements, evacuation routes, and interactions with pupils to develop a student‐centred approach.

By the end of 2018, IsraAID (2018) stated that it had set up an emergency operational plan in 73 schools and trained 11,000 children, teachers, and educational operators. One challenge concerned the trickle‐down effect of this information and the base knowledge of those teachers being trained. Success in each school was largely dependent on the motivation of these trained staff and the willingness to commit to extracurricular work with students to instigate the endeavour. The variability in teacher expertise in DRR and a lack of access to sustained training meant that continued support for the scheme was not uniform across schools. Consequently, some schools developed student groups and led initiatives while others did nothing. This project put pressure on local experts and the limited number of DRR stakeholders who could visit the schools to assist with student training. By 2019, IsraAID had passed on governance of Safer Schools to local leaders.

### Educational DRR from 2018–21

The Smart Schools initiative gained some momentum in some schools up until the end of 2019. With all schools having access to psychosocial support and training, and with at least two teachers per institution having received emergency training, the focus on CCDRR was evident more than had previously been the case. The Ministry of Education sought to carry out school assessments to determine structural needs, and all schools had a disaster plan to help them respond to locally identified hazards. Co‐ordinated and monitored by both the Ministry of Education and IsraAID, it seemed possible that education would achieve the objectives set out by the CRRP (Government of the Commonwealth of Dominica, 2020). One school principal observed that: ‘The school had a disaster manager who was responsible for organising the plan and evacuation routes, [and] that students had been involved in this process and in discussing the potential impacts near to the school’. A teacher reported that, ‘[s]ince the training, the school has organised a group [extracurricular] so that children that are interested in developing an understanding can come and learn from local representatives and participate’. However, another principal highlighted the challenges of initiating the disaster risk plan: ‘Since the training, the teachers involved have been very busy and have not set up the required training with the student groups yet’. One other principal noted that they were struggling to implement the plan due to a lack of expertise: ‘Our staff have attended the training and follow the regulations set by the Ministry of Education, but they are not experts in disaster management’.

From early 2020, the island, just like the rest of the world, was affected by the COVID‐19 pandemic. This presented new unseen risks to Dominica at a time when it was still trying to recover from the impacts of recent hazards. A report by the Organisation of East Caribbean States showed that COVID‐19 led to school closures (throughout early 2020), limiting access to education, and that the unequal distribution of internet technologies and resources within communities restricted student access to online learning (OECS, 2020). This suggests that the impact of the pandemic led to the delayed rollout of the Smart Schools initiative.

The pandemic also delayed the implementation of the Safer Schools initiative, compounded by the departure of IsraAID and a lack of direct management of the scheme in Dominica (IsraAID, 2020). COVID‐19 diverted attention away from the implementation of local school disaster plans towards delivering online education and making possible a return to COVID‐safe schools in September 2020 (Ministry of Education, 2020). One school principal said that ‘since the COVID lockdowns, we have been teaching some students online, but some do not have access to the internet. We have not been able to progress the delivery of any [DRR] teaching’.

This was a common view echoed by many teaching staff and school principals. The reopening of schools was, however, successful in terms of the implementation of psychosocial training, a product of the training received after Hurricane Maria. Staff were encouraged to adopt a student‐centred approach to the return to identify those in need of support and to dedicate the start of term to debriefing on the quarantine period. A core focus of the Safe Schools initiative was to allow students to share information and play while dealing with the emotional impacts of the lockdown and the potential for future change (Ministry of Education, 2020). The restart to the academic year was largely devoted to addressing gaps in student understanding. One principal said that: ‘The key focus has been to continue with repairs to the site caused by Hurricane Maria, and to ensure the students feel safe in the school environment’.

This was a generally held viewpoint across the five secondary schools under review. It was thought that a return to a focus on the DRR curriculum and disaster plans would potentially be factored in by the end of the academic year.

## Discussion

Dominica's educational response to two major disaster events highlights a needs‐response issue that underpins DRR. In the lead up to Tropical Storm Erika in 2015, there was minimal evidence of effective DRE on the island (Carby, 2011). Measures taken to improve community resilience came in the form of the government's CERT scheme and the Red Cross’ Community Disaster Response Teams (CDRT) scheme. While these were bottom‐up approaches, they did not have a specific focus on education. The Red Cross had permanent residence, but its work was largely concerned with the Zika virus outbreak and community resilience in its CDRT scheme (Red Cross, 2018).

Post‐Erika changes in the running of Dominica's Office of Disaster Management and a governmental spotlight on structural improvements meant that proposed modifications to the education system remained a recommendation of the Ministry of Education. The national‐scale impact of Hurricane Maria underlined the need for a more resilient population and to ‘build forward better’. The implementation of the National Resilience Development Strategy 2030 and the subsequent publication of the CRRP (Government of the Commonwealth of Dominica, 2020) aimed to build capacity in communities. Through a developed education curriculum, safer school infrastructure, and a student‐centred approach to DRR, the government followed a pathway towards achieving its wider aim of climate resilience, with a focus on education. This action moved Dominica towards implementing the objectives of the CSSF. The development of bespoke school disaster plans outlined specific needs to improve students’ understanding, to assess the safety of school infrastructure, and to integrate an element of multi‐hazard DRR into the school curriculum.

However, Dominica's post‐Maria vulnerability and declaration to endeavour to be the first ‘climate‐resilient nation’ meant that it was reliant on ODA from nonresident NGOs. This help led to prescribed and needed educational changes and a shift towards addressing the requirements of the CSSF. The short‐term partnership between IsraAID and the Ministry of Education could be considered, though, as ‘conspicuous sustainability’ or ‘conspicuous ODA’ (Grydehoj and Kelman, 2020). Regardless, the implementation of the Safer Schools initiative was a route to meeting much‐needed long‐term DRE needs in Dominica.

One potential issue for the Safer Schools initiative was the need to train teaching staff to disseminate the information. The training, while setting the correct precedent for developing a CCDRR curriculum and emergency response protocols, enjoyed limited direct support from the Office of Disaster Management, a national‐scale disaster risk body, or regional authorities such as the Caribbean Disaster Emergency Management Agency. This unified approach would have accorded teaching staff with the long‐term support necessary to close any gaps in knowledge, especially as not all volunteers had a background in science or geography. For some, therefore, personal experience was the knowledge base, and this did not always reflect the true multi‐hazard risk. A bias towards hydrometeorological hazards outweighed knowledge of geophysical risk in Dominica. Staff commented that their understanding of geophysical risk was linked to those on neighbouring islands, such as the La Soufrière stratovolcano on Saint Vincent or the Kick‐'em‐Jenny ‘live’ submarine volcano in the eastern Caribbean (UWI, 2021). The need for continued support was not being met by the Red Cross, which commented, after Hurricane Maria, that ‘they do not have direct plans in schools for DRR’.

This created the paradox of having the correct approach to meet the CSSF but not the necessary level of ground experience to deliver it. Nevertheless, the Child Friendly Spaces initiative did have some successes in terms of psychosocial support initiated after Tropical Storm Erika, and the UNICEF‐led RTH scheme, implemented after Tropical Storm Erika, allowed teaching staff to manage and support students more effectively following school closures caused by Hurricane Maria and COVID–19. Shillingford, Williams, and Allen (2020) summarised that these approaches were important in building grassroots community support and resilience in local communities. This meant that progress towards Pillar 3 of the CSSF, risk reduction and resilience education, was made in the form of social resilience (Kwok et al., 2016).

A challenge faced by school leaders after Hurricane Maria centred on pressure to reopen schools. The added pressure on the educational system came at a time when Dominica was vulnerable; the staggered return of students was due to school buildings continuing to function as emergency shelters. While the CRRP planned to improve school infrastructure and reduce the use of schools as shelters, meeting the requirements of Pillar 1 of the CSSF, this approach came too late in the wake of Maria. The added pressure created disparities in the provision of education, resulting in non‐examination classes; people who had been forced to migrate and those without online technology were at a disadvantage.

The policy response to COVID‐19 compounded matters, as it extended time away from school and caused delays to the implementation of the Safer Schools and Smart Schools initiatives. Hambleton, Jeyaseelan, and Murphy (2020) show that small island developing states in the Caribbean have a limited capacity and resource base to deal with global pandemics. The restrictions imposed by the Government of the Commonwealth of Dominica may have helped to contain the impact of the virus (Murphy et al., 2020) but they resulted in the loss of essential and continued support from external NGOs with experience of and expertise in integrating DRE reforms. Hambleton, Jeyaseelan, and Murphy (2020) argue that post‐COVID‐19, the government will prioritise economic recovery through tourism.

Given the limited resource base and capacity, prioritising the Safer Schools initiative will thus represent a challenge. Currently, trends in Dominica match those reported in the CSS global baseline survey (Paci‐Green et al., 2020).

## Conclusion

Achieving successful CCDRR has been a challenge for the DRR community around the world. There is a recognised need to develop an integrated approach to DRE to meet the needs of children and vulnerable communities. The HFA and SFA have attempted to promote education through their priorities, although specific targets are not mandated. While they have helped to promote the DRE agenda, it is uncommon for DRE programmes to be implemented until after a disaster. The CSSF has provided governments with guidance on how to improve leadership in education, develop safer school infrastructure, and implement a curriculum that includes DRR with a multi‐hazard emphasis. However, implementing these aims requires a long‐term and sustained focus.

Small island developing states are particularly vulnerable to hazards and have a large youthful population. Dominica is an example of a country whose vulnerability is a product of its culture, history, and exposure to multi‐hazard risk. The development of CCDRR is one tool to help reduce the impact of disasters and build resilience. Dominica's educational response to disaster events reveals the challenges faced by small island developing states in prioritising and implementing change in CCDRR. This case highlights the importance of long‐term policy implementation and the need for continued support to achieve the required successes and identifies the difficulties faced by a short‐term disaster‐reactive policy response. Financial rewards or training may be necessary to motivate teaching staff to sustain initiatives and encourage student participation. Continued local‐scale buy‐in is vital for continuation of CCDRR in the education system, building capacity among children and their communities, and helping the government to meet its goal of a ‘climate resilient nation’.

Dominica has been unfortunate in terms of the challenges presented by successive disasters, but this case points up the need for small island developing states to ensure implementation and prioritisation of DRE to help build continued resilience to multi‐hazard risk in the future. Looking towards ‘building forward better', Shaw, Sakurai, and Oikawa (2021) state that traditional DRR needs to develop new cycles of holistic learning to incorporate resilience‐building, capacity‐building, and risk communication, utilising decision‐making and a participatory methodology. This comprehensive pathway to DRE is one that has been promoted by a number of authors (see, for example, Sakurai and Sato, 2016; Parham et al., 2021; Shaw, Sakurai, and Oikawa, 2021; Kitagawa, 2021) as a means of building a sustainable long‐term approach to CCDRR and implementing the requirements of the CSSF.

## Acknowledgements

This case study was made possible by the assistance of many people. I would like to thank the contributions of all local DRR stakeholders, particularly those representing the Government of the Commonwealth of Dominica, the Ministry of Education, participant secondary schools, the Red Cross, and IsraAID. I would also like to thank the students involved in the longitudinal study who openly contributed between 2013 and 2018.

Finally, I would like to point out that this project received no funding from any external sources.

## Data availability statement

The data that support the findings of this study are available on request from the corresponding author. The data are not publicly available due to privacy or ethical restrictions.

## Endnotes

1 PRISM survey tools use a spatial, visual metaphor to provide a measure of participants’ relative perceptions (Parham et al., 2021).

## References

[disa12536-bib-0001] ACAPS (Assessment Capacities Project) (2017) Dominica: Lessons Learned from Tropical Storm Erika. October. https://www.acaps.org/special-report/dominica-lessons-learned-tropical-storm-erika (last accessed on 4 May 2022).

[disa12536-bib-0002] ACAPS (2018) Dominica: Lessons Learned from Hurricane Maria. January. https://www.acaps.org/sites/acaps/files/products/files/20180123_acaps_dominica_lessons_learned_hurricane_maria_0.pdf (last accessed on 4 May 2022).

[disa12536-bib-0003] Amri, A. , D.K. Bird , K. Ronan , K. Haynes , and B. Towers (2017b) ‘Disaster risk reduction education in Indonesia: challenges and recommendations for scaling up’. Natural Hazards Earth Systems. 17(4). pp. 595–612.

[disa12536-bib-0004] Amri, A. , K. Haynes , D.K. Bird , and K. Ronan (2017a) ‘Bridging the divide between studies on disaster risk reduction education and child-centred disaster risk reduction: a critical review’. Children's Geographies. 16(3). pp. 239–251.

[disa12536-bib-0005] Bandecchi, A.E. , V. Piazzi , S. Morelli , L. Valorie , and N. Casagli (2019) ‘Geo-hydrological and seismic risk awareness at school: emergency preparedness and risk perception evaluation’. International Journal of Disaster Risk Reduction. 40 (November). Article number: 101280. 10.1016/j.ijdrr.2019.101280.

[disa12536-bib-0006] Barclay, J. et al. (2019) ‘Historical trajectories of disaster risk in Dominica’. International Journal of Disaster Risk Science. 10 (June). pp. 149–165.

[disa12536-bib-0007] Benson, C. , E. Clay , F.V. Michael , and A.W. Robertson (2001) Dominica: Natural Disasters and Economic Development in a Small Island State. Disaster Risk Management Working Paper Series No. 2. Disaster Management Facility, World Bank, Washington, DC.

[disa12536-bib-0008] Benson, L. and J. Bugge (2007) Child-led Disaster Risk Reduction: A Practical Guide. Save the Children, London.

[disa12536-bib-0009] Bryman, A. (2016) Social Research Methods. Fifth edition. Oxford University Press, Oxford.

[disa12536-bib-0010] Carby, B. (2011) Caribbean Implementation of the Hyogo Framework for Action, HFA, Mid-term Review. https://www.preventionweb.net/files/18197_203carby.caribbeanimplementationoft.pdf (last accessed on 4 May 2022).

[disa12536-bib-0011] Collymore, J. , B. Carby , P. Rees-Gildea , and G. Thongs (2017) Rapid Review of the Regional Response in the Hurricanes Irma and Maria Events. Final Report. October. https://www.cdema.org/Rapid_Review_of_the_Regional_Response_-_Irma_and_Maria_Events_2017_Final.pdf (last accessed on 4 May 2022).

[disa12536-bib-0012] Cox, R.S. , L. Scannell , C. Heykoop , J. Tobin-Gurley , and L. Peek (2017) ‘Understanding youth disaster recovery: the vital role of people, places, and activities’. International Journal of Disaster Risk Reduction. 22 (January). pp. 249–256.

[disa12536-bib-0013] Delicado, A. , J. Rowland , and S. Fonseca (2017) ‘Children in disaster risk reduction in Portugal: policies, education, and (non) participation’. International Journal of Disaster Risk Science. 8(3). pp. 246–257.

[disa12536-bib-0014] Dondin, F.J.-Y. et al. (2019) ‘The University of the West Indies–Seismic Research Centre Volcano Monitoring Network: evolution since 1953 and challenges in maintaining a state-of-the-art network in a small island economy’. Geosciences. 9(2). Article number: 71. 10.3390/geosciences9020071.

[disa12536-bib-0015] Government of the Commonwealth of Dominica (1998) Plan to Reduce the Vulnerability of School Buildings to Natural Disasters: Dominica. February. https://www.oas.org/cdmp/schools/domweb.htm#4.0%20DETERMINING%20THE%20VULNERABILITY%20OF%20SCHOOL%20BUILDINGS (last accessed on 4 May 2022).

[disa12536-bib-0016] Government of the Commonwealth of Dominica (2001) National Disaster Plan 2001. Prepared by the National Emergency Planning Organisation. https://www.preventionweb.net/publication/national-disaster-plan-commonwealth-dominica (last accessed on 5 May 2022).

[disa12536-bib-0017] Government of the Commonwealth of Dominica (2015) Rapid Damage and Impact Assessment: Tropical Storm Erika – August 27, 2015. 25 September. https://documents1.worldbank.org/curated/en/142861467995411564/pdf/104251-WP-PUBLIC-Rapid-Damage-and-Needs-Assessment-Final-Report-Oct5.pdf (last accessed on 4 May 2022).

[disa12536-bib-0018] Government of the Commonwealth of Dominica (2017) Post-disaster Needs Assessment: Hurricane Maria: September 18, 2017. 15 November. https://reliefweb.int/sites/reliefweb.int/files/resources/dominica-pdna-maria.pdf (last accessed on 4 May 2022).

[disa12536-bib-0019] Government of the Commonwealth of Dominica (2017) ‘Child-friendly schools campaign launched’. Website. https://news.gov.dm/news/56-child-friendly-schools-campaign-launched (last accessed on 5 May 2022).

[disa12536-bib-0020] Government of the Commonwealth of Dominica (2020) Dominica Climate Resilience and Recovery Plan 2020–2030. https://dominica.gov.dm/images/documents/CRRP-Final-042020.pdf (last accessed on 4 May 2022).

[disa12536-bib-0021] Government of the Commonwealth of Dominica (2022) ‘Dominica Meteorological Service’. Website. https://www.weather.gov.dm/ (last accessed on 5 May 2022).

[disa12536-bib-0022] Grydehoj, A. and I. Kelman (2020) ‘Reflections on conspicuous sustainability: creating small island dependent states (SIDS) through ostentatious development assistance (ODA)?’. Geoforum. 116 (November). pp. 90–97.

[disa12536-bib-0023] Hambleton, I.R. , S.M. Jeyaseelan , and M.M. Murphy (2020) ‘COVID-19 in the Caribbean small island developing states: lessons learnt from extreme weather events’. The Lancet Global Health. 8(9). pp. e1114–e1115 32622401 10.1016/S2214-109X(20)30291-6 PMC7332258

[disa12536-bib-0024] Hart, R.A. (1992) Children's Participation. From Tokenism to Citizenship. Innocenti Essays No. 4. March. UNICEF (United Nations Children's Fund) International Child Development Centre, Florence.

[disa12536-bib-0025] Haynes, K. and T. Tanner (2015) ‘Empowering young people and strengthening resilience: youth-centred participatory video as a tool for climate change adaptation and disaster risk reduction’. Children's Geographies. 13(3). pp. 357–371.

[disa12536-bib-0026] Honychurch, L. (1995) The Dominica Story: A History of the Island. Macmillan, London.

[disa12536-bib-0027] Honychurch, L. (2017) In the Forests of Freedom: The Fighting Maroons of Dominica. Papillote Press, London.

[disa12536-bib-0028] Hore, K. , J.C. Gaillard , D. Johnston , and K. Ronan (2018) Child-centred Disaster Risk Reduction. GADRES (Global Alliance for Disaster Risk Reduction in the Education Sector) Research-into-Action Brief. https://www.preventionweb.net/publication/research-action-brief-child-centred-disaster-risk-reduction (last accessed on 5 May 2022).

[disa12536-bib-0029] IFRC (International Federation of Red Cross and Red Crescent Societies) (2021) Integrating CCA and DRR Laws and Policies Towards a Climate-resilient Development: Lessons from the Commonwealth of Dominica. IFRC, Geneva

[disa12536-bib-0030] IsraAID (The Israel Forum for International Humanitarian Aid) (2018) Annual Report 2018. IsraAID, Tel Aviv.

[disa12536-bib-0031] IsraAID (2020) Annual Report 2020. IsraAID, Tel Aviv.

[disa12536-bib-0032] Johnson, V.A. , K.R. Ronan , D.M. Johnston , and R. Peace (2014) ‘Evaluations of disaster education programs for children: a methodological review’. International Journal of Disaster Risk Reduction. 9 (September). pp. 107–123

[disa12536-bib-0033] Kagawa, F. and D. Selby (2012) Disaster Risk Reduction in School Curricula: Case Studies from Thirty Countries. United Nations Children Fund, Geneva, and the United Nations Educational, Scientific and Cultural Organization, Paris.

[disa12536-bib-0034] Kitagawa, K. (2021) ‘Disaster risk reduction activities as learning’. Natural Hazards. 105 (February). pp. 3099–3118.

[disa12536-bib-0035] Knight, P. (2018) ‘UNICEF supporting the return to normality after devastating crises’. United Nations Children's Fund website. 18 September. https://www.unicef.org/easterncaribbean/stories/unicef-supporting-return-normality-after-devastating-crises (last accessed on 4 May 2022).

[disa12536-bib-0036] Knight, V. (2015) ‘Disaster risk reduction education in the Caribbean: policy, practice, and implications for teacher education’. Journal of Eastern Caribbean Studies. 40(3). pp. 187–209.

[disa12536-bib-0037] Kwok, A.H. , E.H. Doyle , J. Becker , D. Johnston , and D. Paton (2016) ‘What is “social resilience”? Perspectives of disaster researchers, emergency management practitioners, and policymakers in New Zealand’. International Journal of Disaster Risk Reduction. 19 (October). pp. 197–211.

[disa12536-bib-0038] Lindsay, J.M. , R.E.A. Robertson , J.B. Sheperd , and S. Ali (2005) Volcanic Hazard Atlas of the Lesser Antilles. Seismic Research Unit, University of the West Indies, St. Augustine.

[disa12536-bib-0039] Ministry of Education (2020) Guidelines and Protocols for the Reopening of Schools: Effective September 2020. Government of the Commonwealth of Dominica, Roseau.

[disa12536-bib-0040] Mitchell, T. , K. Haynes , N. Hall , W. Choong , and K. Oven (2008) ‘The role of children and youth in communicating disaster risk’. Children, Youth and Environments. 18(1). pp. 254–279.

[disa12536-bib-0041] Morais, P. (2019) The Importance of Children's Participation in Disaster Risk Reduction; Facing Environmental Issues. Université Grenoble Alpes, Saint-Martin-d'Hères.

[disa12536-bib-0042] Murphy, M.M. et al. (2020) ‘COVID-19 containment in the Caribbean: the experience of small island developing states’. Research in Globalization. 2 (December). Article number: 100019. 10.1016/j.resglo.2020.100019.

[disa12536-bib-0043] Mutch, C. (2015) ‘The role of schools in disaster settings: learning from the 2010–2011 New Zealand earthquakes’. International Journal of Educational Development. 41 (March). pp. 283–291.

[disa12536-bib-0044] Newnham, E.A. et al. (2019) ‘Tailoring disaster risk reduction for adolescents: qualitative perspectives from China and Nepal’. International Journal of Disaster Risk Reduction. 34 (March). pp. 337–345.

[disa12536-bib-0045] OECS (Organisation of East Caribbean States) (2020) OECS Education Sector Response and Recovery Strategy to COVID-19. OECS Commission, Morne Fortuné.

[disa12536-bib-0046] Ogden, F.L. (2016) ‘Evidence of equilibrium peak runoff rates in steep tropical terrain on the island of Dominica during Tropical Storm Erika, August 27, 2015’. Journal of Hydrology. 542 (November). pp. 35–46.

[disa12536-bib-0047] Paci-Green, R. , A. Varchetta , K. McFarlane , P. Iyer , and M. Goyeneche (2020) ‘Comprehensive school safety policy: a global baseline survey’. International Journal of Disaster Risk Reduction. 44 (April). Article number: 101399. 10.1016/j.ijdrr.2019.101399.

[disa12536-bib-0048] Parham, M. , R. Teeuw , C. Solana , and S. Day (2021) ‘Quantifying the impact of educational methods for disaster risk reduction: a longitudinal study assessing the impact of teaching methods on student hazard perceptions’. International Journal of Disaster Risk Reduction. 52 (January). Article number: 101978. 10.1016/j.ijdrr.2020.101978.

[disa12536-bib-0049] Pasch, R.J. , A.B. Penny , and R. Berg (2019) National Hurricane Center Tropical Cyclone Report: Hurricane Maria (AL152017), 16–30 September 2017. https://www.nhc.noaa.gov/data/tcr/AL152017_Maria.pdf (last accessed on 5 May 2022).

[disa12536-bib-0050] Pasch, R.J. and A.B. Penny (2015) National Hurricane Center Tropical Cyclone Report: Tropical Storm Erika (AL052015), 24–28 August 2015. https://www.nhc.noaa.gov/data/tcr/AL052015_Erika.pdf (last accessed on 5 May 2022).

[disa12536-bib-0051] Paul-Rolle, A. (2014) Disaster Risk Reduction Country Profile: Dominica. September. https://dipecholac.net/docs/files/786-cd-dominica-web.pdf (last accessed on 4 May 2022).

[disa12536-bib-0052] Peek, L. (2008) ‘Children and disasters: understanding vulnerability, developing capacities, and promoting resilience – an introduction’. Children, Youth and Environments. 18(1). pp. 1–29.

[disa12536-bib-0053] Red Cross (2018) Community Disaster Response Teams in Dominica: A Closer Look at Sustainability and Response. https://dipecholac.net/docs/xfiles/1081-CDRT%20Case%20Study%20Dominica.pdf (last accessed on 5 May 2022).

[disa12536-bib-0054] Ronan, K.R. (2015) ‘Progress made with school curricula, education material and relevant training in disaster risk reduction and recovery concepts and practices’. Australian Journal of Emergency Management. 30(1). pp. 8–9.

[disa12536-bib-0055] Ronan, K.R. , K. Crellin , and D.M. Johnston (2012) ‘Community readiness for a new tsunami warning system: quasi-experimental and benchmarking evaluation of a school education component’. Natural Hazards. 61(3). pp. 1411–1425.

[disa12536-bib-0056] Ronan, K.R. and D.M. Johnston (2001) ‘Correlates of hazard education programs for youth’. Risk Analysis. 21(6). pp. 1055–1063. 11824681 10.1111/0272-4332.216174

[disa12536-bib-0057] Ronan, K.R. et al. (2016) ‘Child-centred disaster risk reduction: can disaster resilience programs reduce risk and increase the resilience of children and households?’. Australian Journal of Emergency Management. 31(3). pp. 49–58.

[disa12536-bib-0058] Sakurai, A. , M.B.F. Bisri , R.S. Oktari , and T. Oda (2015) The 11th Years Assessment on School Safety and Disaster Education at the Public Elementary Schools in Banda Aceh after the 2004 Aceh Tsunami: Preliminary Findings. Symposium: National Meeting Bencana Tsunami, Tsunami and Disaster Mitigation Research Center, Banda Aceh, Indonesia, 21–22 December 2015. https://www.researchgate.net/profile/Rina-Oktari/publication/293030025_The_11_th_Years_Assessment_on_School_Safety_and_Disaster_Education_at_the_Public_Elementary_Schools_in_Banda_Aceh_after_the_2004_Aceh_Tsunami_Preliminary_Findings/links/56b5598608aebbde1a77c63e/The-11-th-Years-Assessment-on-School-Safety-and-Disaster-Education-at-the-Public-Elementary-Schools-in-Banda-Aceh-after-the-2004-Aceh-Tsunami-Preliminary-Findings.pdf (last accessed on 5 May 2022).

[disa12536-bib-0059] Sakurai, A. and T. Sato (2016) ‘Promoting education for disaster resilience and the Sendai Framework for Disaster Risk Reduction’. Journal of Disaster Research. 11(3). pp. 402–412.

[disa12536-bib-0060] Sakurai, A. et al. (2018) ‘Exploring minimum essentials for sustainable school disaster preparedness: a case of elementary schools in Banda Aceh City, Indonesia’. International Journal of Disaster Risk Reduction. 29 (August). pp. 73–83.

[disa12536-bib-0061] Sayer, A. (1992) Method in Social Science: A Realist Approach. Second edition. Routledge, London

[disa12536-bib-0062] Siegrist, M. (2013) ‘The necessity for longitudinal studies in risk perception research’. Risk Analysis. 33(1). pp. 50–51. 23231682 10.1111/j.1539-6924.2012.01941.x

[disa12536-bib-0063] Selby, D. and F. Kagawa (2012) Towards a Learning Culture of Safety and Resilience: Technical Guidance for Integrating Disaster Risk Reduction in the School Curriculum. United Nations Children Fund, Geneva, and the United Nations Educational, Scientific and Cultural Organization, Paris.

[disa12536-bib-0064] Serrant, T. (2013) Children, Learning and Chronic Natural Disasters: How Does the Government of Dominica Address Education during Low-intensity Hurricanes? Dissertation submitted to the Graduate Faculty of the School of Education, University of Pittsburgh, in partial fulfilment of the requirements for the degree of Doctor of Philosophy. http://d-scholarship.pitt.edu/20340/ (last accessed on 4 May 2022).

[disa12536-bib-0065] Sharpe, J. (2009) Drills as Part of the Experiential Learning Cycle for Disaster Risk Reduction Education – A Bureaucratic Exercise or Meaning ful Experience? Poster for ‘Disaster Risk Reduction for Natural Hazards: Putting Research into Practice', University College London, London, United Kingdom, November 2009. https://www.ucl.ac.uk/drrconference/posters/JSharpe.pdf (last accessed on 4 May 2022).

[disa12536-bib-0066] Shaw, R. , A. Sakurai , and Y. Oikawa (2021) ‘New realization of disaster risk reduction education in the context of a global pandemic: lessons from Japan’. International Journal of Disaster Risk Science. 12(4). pp. 568–589

[disa12536-bib-0067] Shillingford, A. , N. Williams , and L. Allen (2020) ‘A storm called Erika: lessons from a service-learning community-based psychosocial post disaster response’. Journal of Service-Learning in Higher Education. 10 (January). https://files.eric.ed.gov/fulltext/EJ1242881.pdf (last accessed on 4 May 2022).

[disa12536-bib-0068] Tanner, T. (2010) ‘Shifting the narrative: child-led responses to climate change and disasters in El Salvador and the Philippines’. Children and Society. 24(4). pp. 339–351.

[disa12536-bib-0069] Tanner, T. and K. Haynes (2009) Children as Agents of Change for Disaster Risk Reduction: Lessons from El Salvador and the Philippines. Children in a Changing Climate Research. Working Paper No. 1. April. Institute of Development Studies, Brighton. pp. 1–36.

[disa12536-bib-0070] UNDP (United Nations Development Programme) (2020) Dominica Resettlement Strategy. https://info.undp.org/docs/pdc/Documents/BRB/Draft%20-%20Dominica%20resettlement%20strategy%20v.100915.pdf (last accessed on 4 May 2022).

[disa12536-bib-0071] UNDRR (United Nations Office for Disaster Risk Reduction) (2021) Regional Action Plan for the Implementation of the Sendai Framework for Disaster Risk Reduction 2015–2030 in the Americas and the Caribbean. https://rp-americas.undrr.org/sites/default/files/inline-files/Regional%20Action%20Plan_EN.pdf (last accessed on 4 May 2022).

[disa12536-bib-0072] UNICEF (United Nations Children's Fund) Eastern Caribbean (2017) ‘Dominica schools slowly reopening after devastating hurricane’. Website. 6 March. https://www.unicef.org/easterncaribbean/stories/dominica-schools-slowly-reopening-after-devastating-hurricane (last accessed on 4 May 2022).

[disa12536-bib-0073] UNISDR (United Nations Office for Disaster Risk Reduction) (2005) Hyogo Framework for Action 2005–2015: Building the Resilience of Nations and Communities to Disaster. Website. https://www.unisdr.org/2005/wcdr/intergover/official-doc/L-docs/Hyogo-framework-for-action-english.pdf (last accessed on 4 May 2022).

[disa12536-bib-0074] UNISDR (2009) 2009 UNISDR Terminology on Disaster Risk Reduction. https://www.unisdr.org/files/7817_UNISDRTerminologyEnglish.pdf (last accessed on 4 May 2022).

[disa12536-bib-0075] UNISDR (2013) Programme: Fourth Session of the Global Platform for Disaster Risk Reduction Geneva, Switzerland, 19–23 May 2013. https://www.preventionweb.net/files/32569_en.pdf (last accessed on 5 May 2022).

[disa12536-bib-0076] UNISDR (2015) Chart of the Sendai Framework for Disaster Risk Reduction 2015–2030. https://www.preventionweb.net/publications/view/44983 (last accessed on 4 May 2022).

[disa12536-bib-0077] UNISDR (2017) Comprehensive School Safety. March. https://www.preventionweb.net/publication/comprehensive-school-safety-0 (last accessed on 5 May 2022).

[disa12536-bib-0078] United Nations (2015) Sendai Framework for Disaster Risk Reduction 2015–2030. United Nations, New York, NY.

[disa12536-bib-0079] United Nations General Assembly (1989) Convention on the Rights of the Child. 20 November. https://www.refworld.org/docid/3ae6b38f0.html (last accessed on 4 May 2022). 12344587

[disa12536-bib-0080] UWI (The University of the West Indies) (2021) Kick-'em-Jenny. Website. https://uwiseismic.com/volcanoes/kick-em-jenny/ (last accessed on 4 May 2022).

[disa12536-bib-0081] Van Westen, C.J. (2016) National Scale Landslide Susceptibility Assessment for Dominica. CHARIM – Caribbean Handbook on Risk Information Management. May. World Bank, Washington, DC.

[disa12536-bib-0082] Williams, B.D. and G.R. Webb (2021) ‘Social vulnerability and disaster: understanding the perspectives of practitioners’. Disasters. 45(2). pp. 278–295. 31714606 10.1111/disa.12422

[disa12536-bib-0083] Wilkinson, E. , J. Twigg , and R. Few (2018) Building Back Better: A Resilient Caribbean after the 2017 Hurricanes. Briefing note. January. Overseas Development Institute, London.

[disa12536-bib-0084] Wisner, B. (2006) Let Our Children Teach Us: A Review of the Role of Education and Knowledge in Disaster Risk Reduction. Books for Change, Bangalore.

[disa12536-bib-0085] Wisner, B. , J.C. Galliard , and I. Kelman (2012) The Routledge Handbook of Hazards and Disaster Risk Reduction. Routledge, London.

[disa12536-bib-0086] Wisner, B. et al. (2018) ‘Communication with children and families about disaster: reviewing multi-disciplinary literature 2015–2017’. Current Psychiatry Reports. 20(9). pp. 42–47. 30094701 10.1007/s11920-018-0942-7

[disa12536-bib-0087] World Bank (2015) ‘Dominica lost almost all its GDP due to climate change’. Website. 1 December. https://www.worldbank.org/en/news/feature/2015/12/01/dominica-lost-almost-all-gdp-climate-change (last accessed on 4 May 2022).

[disa12536-bib-0088] World Bank (2018) ‘World development indicators’. Databank. https://databank.worldbank.org/reports.aspx?source=2&country=DMA (last accessed on 4 May 2022).

